# Characteristics and Emerging Trends in Research on Rehabilitation Robots from 2001 to 2020: Bibliometric Study

**DOI:** 10.2196/42901

**Published:** 2023-05-31

**Authors:** Ying Zhang, Xiaoyu Liu, Xiaofeng Qiao, Yubo Fan

**Affiliations:** 1 School of Biological Science and Medical Engineering Beihang University Beijing China; 2 Key Laboratory of Biomechanics and Mechanobiology of Ministry of Education Beihang University Beijing China; 3 Beijing Advanced Innovation Center for Biomedical Engineering Beihang University Beijing China; 4 Institute of Information and Artificial Intelligence Technology Beijing Academy of Science and Technology Beijing China; 5 State Key Laboratory of Virtual Reality Technology and Systems Beihang University Beijing China

**Keywords:** rehabilitation robot, bibliometric analysis, interdisciplinary research, co-occurrence analysis, co-citation analysis, rehabilitation

## Abstract

**Background:**

The past 2 decades have seen rapid development in the use of robots for rehabilitation. Research on rehabilitation robots involves interdisciplinary activities, making it a great challenge to obtain comprehensive insights in this research field.

**Objective:**

We performed a bibliometric study to understand the characteristics of research on rehabilitation robots and emerging trends in this field in the last 2 decades.

**Methods:**

Reports on the topic of rehabilitation robots published from January 1, 2001, to December 31, 2020, were retrieved from the Web of Science Core Collection on July 28, 2022. Document types were limited to “article” and “meeting” (excluding the “review” type), to ensure that our analysis of the evolution over time of this research had high validity. We used CiteSpace to conduct a co-occurrence and co-citation analysis and to visualize the characteristics of this research field and emerging trends. Landmark publications were identified using metrics such as betweenness centrality and burst strength.

**Results:**

Through data retrieval, cleaning, and deduplication, we retrieved 9287 publications and 110,619 references cited in these publications that were on the topic of rehabilitation robots and were published between 2001 and 2020. Results of the Mann-Kendall test indicated that the numbers of both publications (*P*<.001; *S_t_*=175.0) and citations (*P*<.001; *S_t_*=188.0) related to rehabilitation robots exhibited a significantly increasing yearly trend. The co-occurrence results revealed 120 categories connected with research on rehabilitation robots; we used these categories to determine research relationships. The co-citation results identified 169 co-citation clusters characterizing this research field and emerging trends in it. The most prominent label was “soft robotic technology” (the burst strength was 79.07), which has become a topic of great interest in rehabilitative recovery for both the upper and lower limbs. Additionally, task-oriented upper-limb training, control strategies for robot-assisted lower limb rehabilitation, and power in exoskeleton robots were topics of great interest in current research.

**Conclusions:**

Our work provides insights into research on rehabilitation robots, including its characteristics and emerging trends during the last 2 decades, providing a comprehensive understanding of this research field.

## Introduction

The past 2 decades have seen rapid, vast development of robots for rehabilitation. Rehabilitation robots are representative of advanced modern rehabilitation devices; they are automatically operated machines used to treat patients with impaired motor function [1-5]. Research on rehabilitation robots is interdisciplinary, spanning fields such as computer science, mechanical engineering, and medicine. Some publications, mainly in engineering-related journals, focus on the design of robotic structures and mechanics [6-10]. Others focus on control strategies and biomedical signals applied to human-computer interactions [11]. Moreover, a great deal of literature has emphasized the application of rehabilitation robots in treatment and their clinical effectiveness [12]. The variety in this field thus makes it is a great challenge for researchers to understand the overall development of rehabilitation robots and emerging trends in research.

Literature reviews provide researchers with a comprehensive understanding of particular areas of research [13]. Most existing review papers are written from the perspective of the reviewers, which may be constrained by the subjectivity of their evaluation and their cognitive approach [9]. In addition, review papers may lack an overall view of the different research fields and their relationships in their statistical analyses. Without quantitative methods, it is extremely difficult to obtain an objective and comprehensive understanding of a specific research field [14-18].

Recently, improvements in computer and information science have enhanced bibliometric analysis and allowed an intensive interpretation of emerging trends in single and multiple research fields [19-21]. The visualization of collaborative networks and research themes helps researchers understand the current state of specific research areas and their future trends [22,23]. The intellectual structure of research and emerging trends can be characterized by co-occurrence and co-citation clustering [24]. In recent years, a combined use of symbolic (ie, linguistic) and numeric information has helped identify hot topics and research trends in various research fields [25,26]. There have been some bibliometric studies on topics related to rehabilitation conducted from the perspectives of clinical treatment and engineering technology. Feng et al [27] pioneered a bibliometric analysis that provided an understanding of international research trends in stroke rehabilitation treatment. Another report, on the topic of exercise for stroke rehabilitation, revealed hotspots and emerging trends in this research field [28]. Based on co-occurrence and co-citation networks, burst keywords and clustering of similar research topics were used to identify and understand exercise interventions for stroke between 2001 and 2021. A more recent study used bibliometric analysis to predict that novel technologies, such as virtual reality, robotic interfaces, and brain-computer interfaces, will remain hotspots in rehabilitation treatment in the coming years [29]. A report describing relationships within the scientific literature on the application of virtual and augmented reality in medicine revealed the high potential and diversity of applications of virtual reality in stroke rehabilitation [30]. Robotic technology has been considered an important means of technical assistance and an effective approach for the rehabilitation of motor function. However, to our knowledge, no bibliometric report has yet determined the characteristics of research on the use of robots in rehabilitation and emerging trends in this field. 

In this study, we performed a bibliometric analysis, including a co-occurrence and co-citation network analysis, of research conducted between 2001 and 2020 on robots for rehabilitation. Our aim was to understand developments in this research field and identify emerging trends.

## Methods

### Data

The bibliometric data used in this study were derived from scientific literature indexed by the Web of Science (WoS) Core Collection as of July 28, 2022. A comprehensive search strategy was used to meet the requirements for data coverage. This strategy involved both index terms and keywords, including truncation, proximity, and phrases. Terms such as “rehabilitation robot” were searched for as “rehabili* robot*” to identify all related terms. Document types were limited to “article” and “meeting,” excluding documents classified as “review” papers. The records included basic attributes of the documents, such as publication time, author, institution, country, and cited references; these were used to form a database that was used for the subsequent analysis.

### Tools and Procedure

In this paper, CiteSpace, a Java application developed by Chen [18], was used to generate co-occurrence and co-citation networks and analyze their characteristics. This method has been widely used in scientometric research [28,29,31,32]. The time slicing was from 2001 to 2020 with 1 year per slice. The sources that were searched included titles, abstracts, author keywords, and “keywords plus,” which are automatically generated by the WoS. The node types included the category, cited journal, references, and keywords. The selection criterion was that the publication was one of the top 50 for highest number of citations in each time slice.

### Overall Trend Analysis

A Mann-Kendall test was used to assess whether the literature data, including publications and citations, increased year over year, and whether trends were statistically significant. For each comparison pair (publications or citations in 2 adjacent years), we assigned a score of +1 if the latter value was greater than the former value and a score of –1 if the latter value was lower than the former value. All scores were then summed to calculate the test statistic, *S_t_*. A positive *S_t_* value means that the trend is increasing, and a negative *S_t_* value means that the trend is decreasing.

### Co-occurrence and Co-citation Clustering

A co-occurrence analysis was performed to observe the relationship between shared words in the literature. The frequency of word occurrence is associated with the underlying themes. In this analysis, a co-occurrence clustering network was generated to examine changes in specific topics in research on the use of robots in rehabilitation [33,34]. A co-citation analysis was performed to complete the comparative analysis by detecting emerging topics from the selected bibliographic data. This analysis assumed that 2 papers appearing in the reference list of a third related paper form part of the structure of intellectual knowledge related to those papers in terms of networks of cocited references [35,36]. Co-citation clusters were obtained from the synthesized network. For each cluster, labels were automatically identified from noun phrases and index terms of papers that made citations in each cluster. In our study, a log-likelihood ratio (LLR) algorithm was used to determine ranks for the labels for each cluster, since it has been found that LLR algorithms usually give the best results in terms of uniqueness and coverage [37,38]. More details on the LLR algorithm can be found in Multimedia Appendix 1 [5,24,38-41].

### Metrics

The co-occurrence network and co-citation networks were mainly characterized by 2 metrics, betweenness centrality (BC) and burst strength (BS). BC was used to identify pathways between different thematic clusters. This computation was based on a fast algorithm introduced by Brandes [42] that allows measuring the extent to which a node is in the middle of a path connecting other nodes in the network [43]. BS was used as an indicator of dramatic increases in publications in a short period of time. In this study, BS in each cluster was determined using an algorithm reported by Kleinberg [44] that is elaborated in detail in Multimedia Appendix 2 [44,45]. Furthermore, we used the modality (Q) and the mean silhouette (S) to indicate the quality of co-citation cluster networks [24,46]. Q, ranging from 0 to 1, is an indicator of the quality of modular organization in a network. Q>0.3 indicates that the organization of the network is superior. S, ranging from 0 to 1, is an indicator of the homogeneity of a network. S>0.5 represents valid clustering in the network; S>0.7 indicates high homogeneity of the network.

## Results

### Overall Literature Landscape

**Figure 1:**
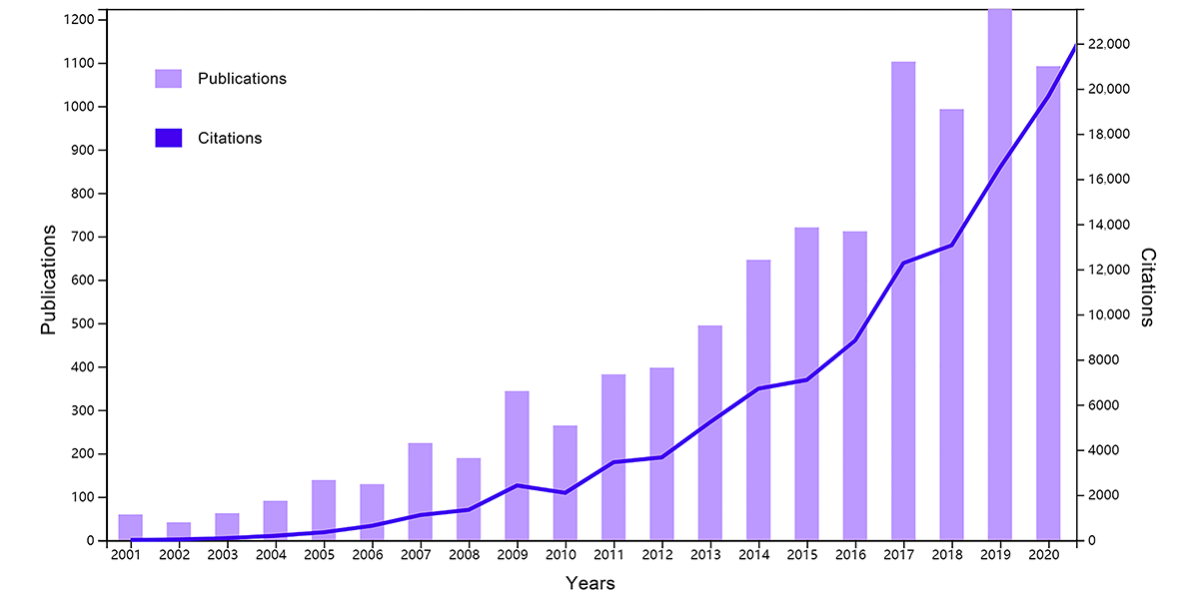
Web of Science–indexed publications from 2001 to 2020 on the topic of robots for rehabilitation and citations in these publications.

After data retrieval, cleaning, and deduplication, we retrieved a total of 9287 publications and 110,619 cited references in the field of research on robots for rehabilitation that were published from 2001 to 2020; we used these data to form a database (Multimedia Appendix 3). The number of published and cited papers on rehabilitation robots per year from 2001 to 2020 is shown in Figure 1. The Mann-Kendall test was used to assess yearly trends. The results indicated that the numbers of both publications (*P*<.001; *S_t_*=175.0) and citations (*P*<.001; *S_t_*=188.0) related to rehabilitation robots have exhibited a significantly increasing yearly trend in the last 2 decades. In the past 10 years (2011-2020), there is an obvious, fast-growing trend in the number of citations. The growth rate of citations is faster than that of publications.

### Co-occurrence Analysis

**Figure 2:**
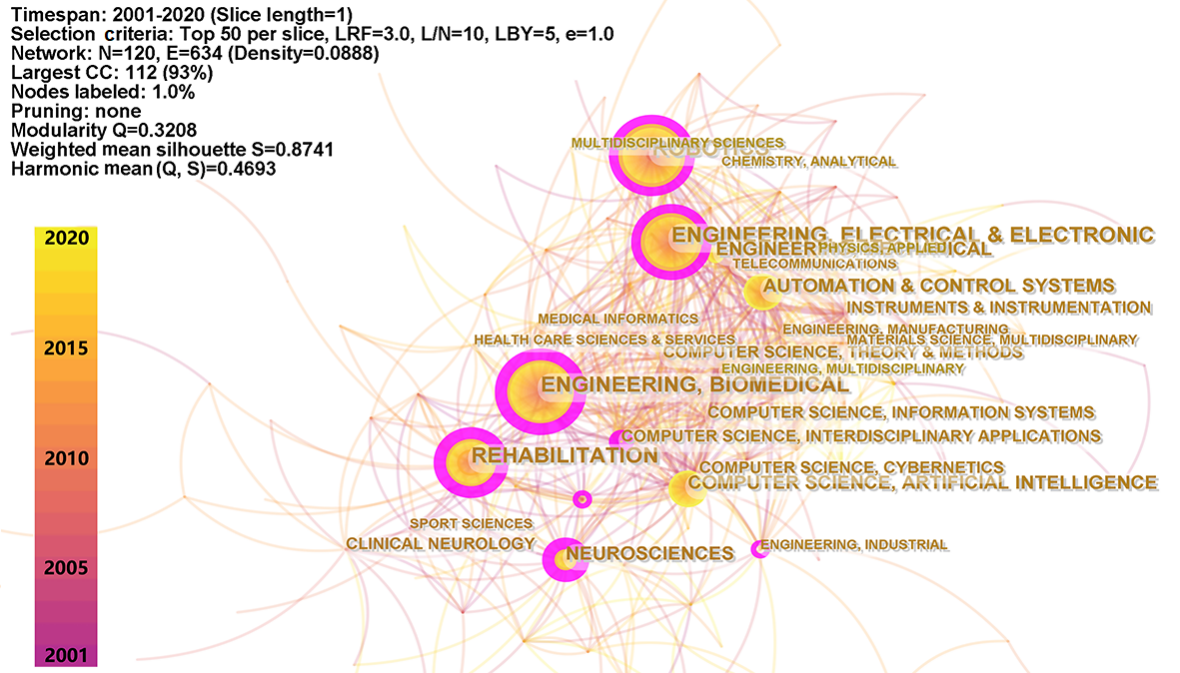
Co-occurrence network of publications on rehabilitation robots published from 2001 to 2020. CC: co-citation; E: edge.; e: equivalency; L/N: link/node; LBY: look back years; LRF: link retaining factor; N: node; Q: modality; S: weighted mean silhouette.

Each publication indexed in the WoS is assigned to one or more categories. Based on the classification of categories in the database [47], publications on rehabilitation robots have been distributed in 120 categories during the past 2 decades. We mapped a co-occurrence network of the internal connections between categories (Figure 2). The results show that the top four categories for publications on rehabilitation robots were (1) *robotics*, (2) *electrical and electronic engineering*, (3) *biomedical engineering*, and (4) *automation* *and* *control system*. The major categories in medical fields were *rehabilitation*, *neurosciences*, *clinical neurology*, *sport sciences*, and *medical informatics*. Table 1 lists the metrics for category co-occurrence in detail. The categories with high BC are considered hubs in the co-occurrence network that link different categories in research on rehabilitation robots. The categories with high BS are considered to have generated the most interest in the past 2 decades.

**Table 1:** Prominent co-occurrence categories in publications related to rehabilitation robots (2001-2020).

Publications, n	Burst strength	Betweenness centrality	Categories
3334	N/A^a^	0.14	*Robotics*
2498	N/A	0.16	*Engineering, electrical and electronic*
2285	26.01	0.21	*Engineering, biomedical*
1822	N/A	0.25	*Rehabilitation*
1557	N/A	0.06	*Automation and control systems*
1412	19.36	0.09	*Computer science, artificial intelligence*
830	N/A	0.24	*Neurosciences*
716	N/A	0.07	*Engineering, mechanical*
454	N/A	0.02	*Computer science, information systems*
447	N/A	0.04	*Computer science, cybernetics*
416	11.54	0.06	*Computer science, theory, and methods*
333	20.02	0.27	*Computer science, interdisciplinary applications*
322	N/A	0.07	*Clinical neurology*
278	N/A	0.04	*Instruments and instrumentation*
219	N/A	0.07	*Engineering, multidisciplinary*
219	N/A	0.03	*Engineering, manufacturing*
193	N/A	0.05	*Materials science, multidisciplinary*
185	6.13	0	*Sport sciences*
179	N/A	0.02	*Medical informatics*
172	N/A	0.01	*Telecommunications*
156	8.97	0	*Multidisciplinary sciences*
145	N/A	0.04	*Physics, applied*
127	N/A	0.12	*Engineering, industrial*
114	N/A	0.01	*Chemistry, analytical*
102	N/A	0.01	*Health care sciences and services*
95	10.79	0.01	*Biophysics*
85	N/A	0.02	*Mathematical and computational biology*
81	N/A	0	*Mechanics*
78	14.93	0.12	*Medicine, research and experimental*
78	4.00	0.03	*Orthopedics*

^a^N/A: not applicable.

### Co-citation Analysis

**Figure 3:**
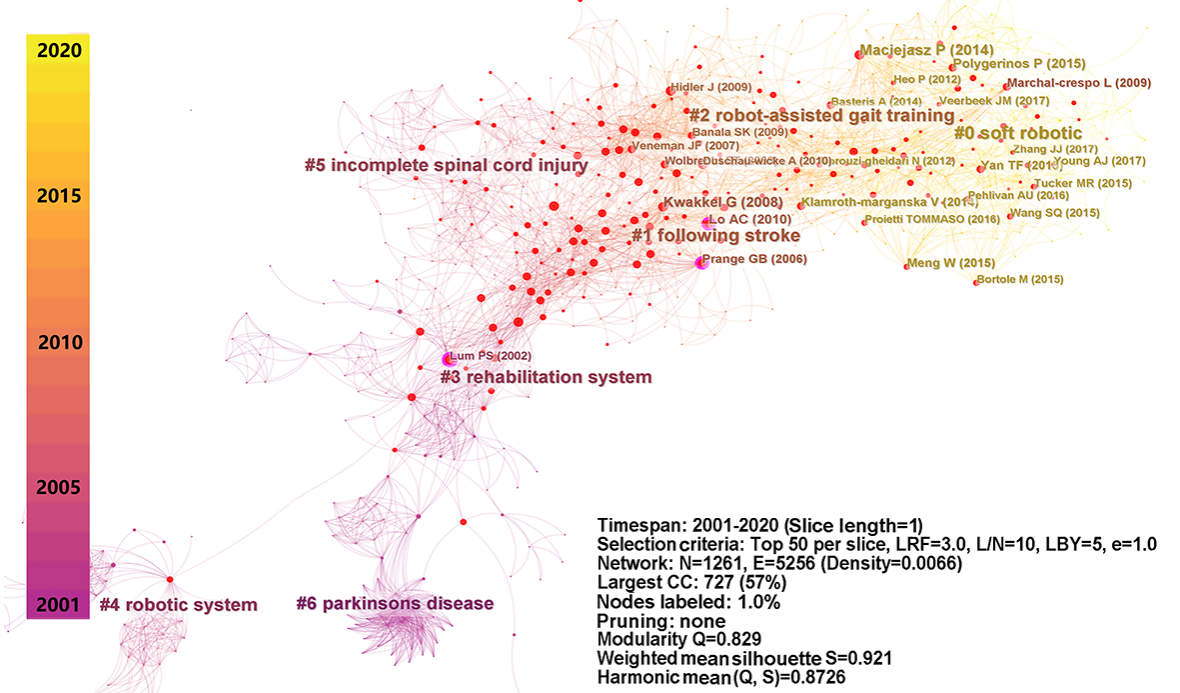
Network of co-citation clusters of publications on rehabilitation robots published from 2001 to 2020. CC: co-citation; E: edge.; e: equivalency; L/N: link/node; LBY: look back years; LRF: link retaining factor; N: node; Q: modality; S: weighted mean silhouette.

Based on the literature data, a network of co-citation clusters was constructed and visualized (Figure 3) that contained 1261 nodes and 5256 links. The nodes represent cited papers that have been labeled with representative authors and publication years. A total of 169 co-citation clusters of publications related to rehabilitation robots were identified and automatically labeled. Research trends are characterized by clusters of papers cited by corresponding research publications. In the network, Q was 0.892 and mean S was 0.921, indicating that the network was reliable and had high quality for co-citation structure. In particular, 6 prominent clusters were identified as representing important research themes; these are enumerated in detail in Table 2, which includes information on the size, S value, year, duration, and label of the top 6 clusters. Size refers to the number of cited papers in the cluster, which determines the volume of the cluster. A high S value denotes good homogenization of nodes in each cluster. In this analysis, S was >0.85 for the top 6 clusters, indicating that they had high credibility [48].

**Figure 4:**
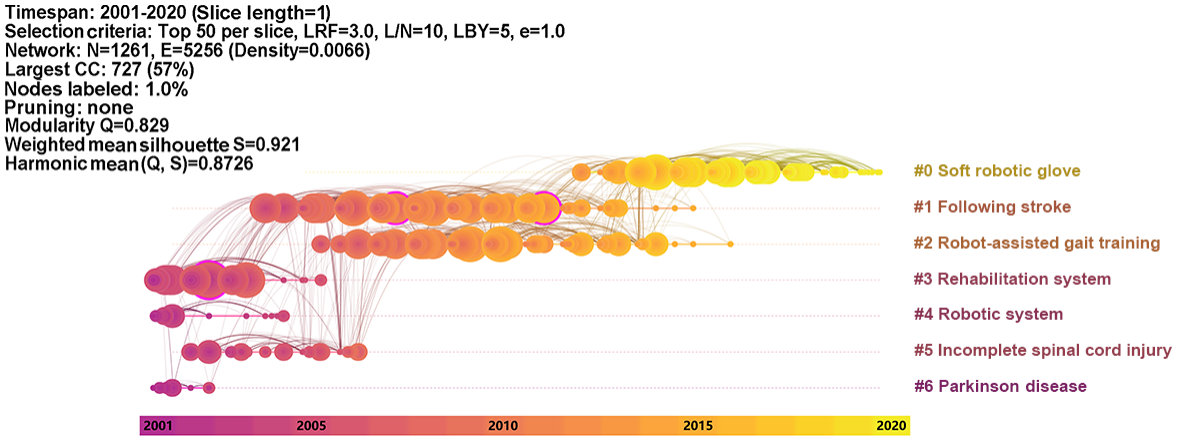
Timeline view of co-citation clusters of publications from 2001 to 2020 on rehabilitation robots. The publication year is displayed horizontally and prominent labels identified from each cluster are displayed vertically. CC: co-citation; E: edge.; e: equivalency; L/N: link/node; LBY: look back years; LRF: link retaining factor; N: node; Q: modality; S: weighted mean silhouette.

Further, we used the timeline view to visualize the evolution of the publications (Figure 4). In this view, each timeline represents a specific research trend, and the evolution of the specific research is determined by the release of milestone publications based on the timeline. It is observed that cluster 0 (*soft robotic*) is currently a research hotspot, while cluster 1 (*following stroke*) and cluster 2 (*robot-assistant gait training*) have gradually lost attention. Recently, few reports have been published on topics related to the robotic treatment of patients with spinal cord injury or Parkinson disease.

**Table 2:** Prominent co-citation clusters related to rehabilitation robots in research published from 2001 to 2020.

Cluster	Size, n	Silhouette	Year	Duration	Labels
0	186	0.877	2017	2009 to 2019	soft robotic; hand rehabilitation; chronic stroke; stroke survivor; stroke patient; pilot study; robotic rehabilitation; daily living; exoskeleton robot; gait rehabilitation
1	122	0.854	2008	2001 to 2012	following stroke; chronic stroke; stroke patient; pilot study; chronic stroke patient; robotic device; stroke rehabilitation; motor learning; motor recovery
2	98	0.892	2011	2003 to 2013	robotic-assistant gait rehabilitation; active participation; gait training; post-stroke early neurorehabilitation; cable-driven locomotor training system; human spinal cord injury; packet loss
3	83	0.967	2002	1996 to 2003	stroke patient; chronic stroke; rehabilitation system; using actuator; robotic therapy; robot-assisted movement training; conventional therapy technique; follow-up result; potential recovery
4	58	1.000	2002	1996 to 2005	robotic system; intelligent sweet home; welfare-oriented service; effective intention reading; visual servoing; human-friendly man-machine interaction unit; novel type rehabilitation; wheelchair-based robotic arm; effective intention reading
5	56	0.946	2005	1999 to 2005	incomplete spinal cord injury; joint kinematics; ankle-foot orthoses; muscle activation; gait training; measuring human training skill; robot control algorithm; robotic-assisted treadmill training

### Publications With High Metrics

BC and BS are two important metrics in the co-citation analysis. Table 3 lists the papers with the top 10 BC ratios of all nodes in the network. There are 4 papers (ranked 1 to 4) that should be regarded as landmark publications in the field of research on rehabilitation robots. The publication by Lum et al [49] was identified as having the highest BC (0.15) and has been cited in a variety of research fields, including robotics (444 citations), computer science (427 citations), neuroscience (350 citations), and rehabilitation (342 citations). Table 4 lists the papers in the network with the top 10 BS. A prominent publication on soft-structured gloves (BS was 79.07) for home-based rehabilitation represents the current hot topic in research on robotic rehabilitation.

**Table 3:** Top 10 publications with highest betweenness centrality.

Rank	Betweenness centrality	Authors	Year	Publication title
1	0.15	Lum et al [49]	2002	Robot-assisted movement training compared with conventional therapy techniques for the rehabilitation of upper-limb motor function after stroke
2	0.12	Prange et al [50]	2006	Systematic review of the effect of robot-aided therapy on recovery of the hemiparetic arm after stroke
3	0.10	Lo et al [51]	2010	Robot-assisted therapy for long-term upper-limb impairment after stroke
4	0.10	Song et al [52]	1999	KARES: Intelligent wheelchair-mounted robotic arm system using vision and force sensor
5	0.07	Krebs et al [53]	1998	Robot-aided functional imaging: application to a motor learning study
6	0.06	Reinkensmeyer et al [54]	1999	Guidance-based quantification of arm impairment following brain injury: A pilot study
7	0.05	Maciejasz et al [1]	2014	A survey on robotic devices for upper limb rehabilitation
8	0.05	Fasoli et al [55]	2003	Effects of robotic therapy on motor impairment and recovery in chronic stroke
9	0.04	Ferraro et al [56]	2003	Robot-aided sensorimotor arm training improves outcome in patients with chronic stroke
10	0.04	Stienen et al [57]	2009	Self-aligning exoskeleton axes through decoupling of joint rotations and translations

**Table 4:** Top-10 publications with highest burst strength.

Rank	Burst strength	Authors	Year	Duration	Publication title
1	79.07	Maciejasz et al [1]	2014	2015 to 2020	A survey on robotic devices for upper limb rehabilitation
2	42.36	Polygerinos et al [58]	2015	2016 to 2020	Soft robotic glove for combined assistance and at-home rehabilitation
3	39.50	Meng et al [59]	2015	2017 to 2020	Recent development of mechanisms and control strategies for robot-assisted lower limb rehabilitation
4	34.94	Klamroth-Marganska et al [60]	2014	2015 to 2020	Three-dimensional, task-specific robot therapy of the arm after stroke: a multicentre, parallel-group randomised trial
5	33.88	Young et al [61]	2017	2018 to 2020	State-of-the-art and future directions for lower limb robotic exoskeletons
6	33.38	Yan et al [62]	2015	2016 to 2020	Review of assistive strategies in powered lower-limb orthoses and exoskeletons
7	30.65	Veerbeek et al [63]	2017	2018 to 2020	Effects of robot-assisted therapy for the upper limb after stroke: a systematic review and meta-analysis
8	29.14	Tucker et al [9]	2015	2017 to 2020	Control strategies for active lower extremity prosthetics and orthotics: a review
9	27.42	Zhang et al [64]	2017	2018 to 2020	Human-in-the-loop optimization of exoskeleton assistance during walking
10	25.58	Awad et al [65]	2017	2018 to 2020	A soft robotic exosuit improves walking in patients after stroke

## Discussion

### Principal Findings

In this study, we performed a bibliometric analysis of research on rehabilitation robotics published in the last 2 decades; we obtained insights on emerging trends in this research field. Our analysis indicates that the emergence of new technologies, such as virtual reality, brain-computer interfaces, and intelligent sensing, is advancing the development of rehabilitation robotics. Robots with a flexible structure are currently attracting great attention in robot design.

### Literature Data

Literature (9287 publications in total) retrieved from the WoS database between 2001 and 2020 shows a continuous increase in research interest in rehabilitation robotics. We excluded review papers from the literature data because they might have interfered with the bibliometric analysis; compared with original-research papers, review articles are more likely to be identified as prominent publications because they are usually highly cited by other researchers. However, most review papers are not associated with technical keywords. Even if some review papers provide technical keywords, they lag behind the publication date of the paper. Consequently, specific technologies identified as being important and having an increasing trend likely do not match the period of their actual emergence. For this reason, we excluded review papers from our document retrieval.

### Research Characteristics

Based on the literature data, we performed co-occurrence and co-citation analyses to characterize emerging trends in research on rehabilitation robots. The co-occurrence analysis of categories showed that a majority of articles on rehabilitation robots are published in fields related to engineering, such as robotics, electrical and electronic engineering, and biomedical engineering. The development of rehabilitation robotics has been going on for many years, and technological progress will continue in the coming decades. Newly emerging engineering technologies (eg, virtual reality, brain-computer interfaces, and intelligent sensing) are key drivers of advances in the development of rehabilitation robotics [30,66,67]. For example, many commercially available robots can perform interactive tasks with multisensory involvement based on virtual reality rendering [68]. Recent studies have reported that brain-computer interfaces based on electroencephalography can help survivors of severe spinal cord injuries regain activities of daily living [69]. Also, intelligent sensory systems facilitate more natural and efficient interaction between rehabilitation robots and patients [70]. In medical research, a majority of articles on rehabilitation robots have been published in categories such as rehabilitation, clinical neurology, sports science, and medical informatics. These publications are closely related to the clinical application of robots in therapy and assessment for patients with stroke, spinal cord injury, and traumatic brain injury. Clinical studies generally agree that rehabilitation robots provide customized, task-oriented, prolonged, intensive, standardized, and repeatable training for patients [50], although some reports have suggested that rehabilitation robots do not exhibit significantly superior clinical effects compared to conventional treatments [49]. It is believed that robot-aided rehabilitation will have improved clinical effectiveness if novel technologies continue to be incorporated [71].

### Emerging Trends

The co-citation analysis identified 169 clusters, allowing us to characterize and interpret the structure and dynamics of co-citation in research on rehabilitation robots. Specifically, we identified 7 prominent clusters that represent important research themes. The largest cluster (cluster 0; Multimedia Appendix 4) included the following labels: *soft robotic*, *hand rehabilitation*, *chronic stroke*, *exoskeleton robot*, and *gait rehabilitation*. This cluster emerged between the years 2009 and 2019. Recently, soft robotics in rehabilitation have become a novel branch of robotics; they are characterized by compliant and flexible materials (eg, rubber, silicone, elastomers, and nylon 6) [72]. Compared to robots with rigid materials, soft robots are more comparable to soft biological tissues and organs, which allows increased flexibility and adaptability for use by patients [58]. Such flexible systems are highly compliant and able to perform a range of natural and flexible interactions, facilitating a range of natural and friendly movements during training; their safety and low risk also allow for home-based rehabilitation procedures. One of the most prominent publications in this cluster was a work by Polygerinos et al [58] that reported a novel soft robotic glove for hand rehabilitation. This robotic design is expected to provide a clear path to develop soft robots for both upper and lower limb rehabilitation [73]. Another prominent cluster (cluster 1; Multimedia Appendix 5) covered the topic of diseases or impairments treated by robots. Stroke was identified in this cluster, suggesting that rehabilitation robots are being used more extensively in the treatment of stroke, especially chronic stroke. Robotic rehabilitation therapy can deliver high-dosage and high-intensity training, making it useful for patients with motor disorders caused by stroke in the chronic stage [74]. Although several clinical applications have focused on motor recovery in patients with acute stroke, this use is highly risky among this population, because it interferes with conventional treatment [75]. Additionally, short-term robotic rehabilitation makes only a small contribution to clinical effectiveness. A prominent publication reported that robot-assisted therapy did not significantly improve motor function after a 12-week course of treatment but had a therapeutic effect after 36 weeks [51]. Cluster 2 (Multimedia Appendix 6) was mainly related to gait rehabilitation with robot assistants, which is a cutting-edge technology used in rehabilitation after neurological injuries and for conditions like spinal cord injury, brain injury, stroke, multiple sclerosis, Parkinson disease, and cerebral palsy [76]. Robot assistants allow patients to perform effective motion training for upper and lower limb recovery [77]. Cluster 3 concentrated on clinical treatment assisted by rehabilitation robots, highlighting chronic stroke and movement training. Cluster 4 focused on human-machine interaction and indicated that effective intention-reading by robots and visual interaction had benefits that included friendlier interaction. Treatment of spinal cord injuries healed by robots was the principal concern of cluster 5, which examined orthoses, muscle activation, joint kinematics, and treadmill training as key considerations in robot design. We will omit discussion of cluster 6, because the identified duration of these clusters mainly ranged from 1999 to 2005, meaning that they no longer represent hot topics in current research.

### Limitations

Limitations of this bibliometric analysis include, first, that it was based on literature data retrieved only from the WoS database. This database might not include publications that were present in other databases, such as PubMed, Google Scholar, and Scopus, meaning that they were missing from our analysis. The main reason for selecting the WoS was that this database is designed to allow citation analysis and provides more extensive information [73,78,79]. Reference links in the WoS allowed us to identify all publications cited as similar research. As one of the most important literature databases, WoS includes all high-influence publications that contribute to the intellectual structure of this research field and to emerging trends. Therefore, the publications that were potentially missing from our literature database likely would have had little impact on the bibliometric results. A second limitation of this study is related to the categories in our co-occurrence analysis: there was no consistent classification method for subject categories. It is common for an article to be included in different categories across literature databases [80]. Here, we used the categories of the WoS to classify the retrieved publications because this method is commonly used in bibliometric analyses.

### Conclusions

This study identified prominent literature on the use of robots in rehabilitation through bibliometric analysis. We visualized and characterized co-occurrence and co-citation networks of publications in this research field, providing insights into the characteristics of the research and emerging trends over the last 2 decades. Our co-occurrence analysis showed that the emergence of new engineering technologies (eg, virtual reality, brain-computer interfaces, and intelligent sensing) advances the development of rehabilitation robotics. Our co-citation analysis indicates that flexible-structure robots are currently gaining wide attention for rehabilitative applications. Rehabilitation robots have been used most extensively in the treatment of chronic stroke. It is foreseeable that research on rehabilitation robots in the coming years will enjoy explosive growth that will promote extensive applications in improving motor function and quality of life in human patients.

## Appendix

Multimedia Appendix 1 Log-likelihood ratio (LLR) algorithm.

Multimedia Appendix 2 The burst detection algorithm and its interpretation.

Multimedia Appendix 3 Database.

Multimedia Appendix 4 Top 20 cited references in cluster #0.

Multimedia Appendix 5 Top 20 cited references in cluster #1.

Multimedia Appendix 6 Top 20 cited references in cluster #2.

## Abbreviations

BC: betweenness centrality
BS: burst strength
LLR: log-likelihood ratio
Q: modality
S: mean silhouette
WoS: Web of Science

## Data Availability

The data sets generated during and/or analyzed during this study are available from the corresponding author on reasonable request.
