# Supplementary material for: Characteristics and Emerging Trends in Research on Rehabilitation Robots from 2001 to 2020: Bibliometric Study
Source: J Med Internet Res. 2023 May 31;25:e42901. doi: 10.2196/42901 (PMC10267796; doi:10.2196/42901)
Supplement: Multimedia Appendix 1 [file jmir_v25i1e42901_app1.docx]

**Log-likelihood ratio (LLR) algorithm**

Usually, cluster labels are usually ranked by three different algorithms [5, 24, 38], including the tf*idf [39], the log-likelihood ratio (LLR) tests [40], and the mutual information (MI) [41]. We selected the LLR test to rank cluster labels, since it has been found that that LLR usually gives the best result in terms of the uniqueness and coverage. The LLR tests are based on the idea that statistical hypotheses can be believed to specify subspaces of the space described by the unknown parameters of the statistical model being used. The probability that a given experimental outcome described by *k*_1_, *k*_2_, ..., *k*_n_ will be observed for a given model described by a number of parameters. *p*_1_, *p*_2_, ..., *p*_n_, is called the likelihood function for the model and is written as:

$$H(p_{1}, p_{2},\ldots; k_{1}, k_{1},\ldots)$$

where all arguments of *H* left of the semicolon are model parameters, and all arguments right of the semicolon are observed values. In the continuous case, the probability is replaced by a probability density. With binomial and multinomials, we only deal with the discrete case.

The likelihood ratio for a hypothesis is the ratio of the maximum value of the likelihood function over the subspace represented by the hypothesis to the maximum value of the likelihood function over the entire parameter space. That is,

$$\lambda=\frac{\max_{\omega\in\Omega_{0}}H(p;k)}{\max_{\omega\in\Omega}H(p;k)}$$

where Ω is the entire parameter space and Ω_0_ is the particular hypothesis being tested.

The particularly important feature of likelihood ratios is that the quantity - 2log*λ* is asymptotically *χ*^2^ distributed with degrees of freedom equal to the difference in dimension between Ω and Ω_0_. Importantly, this asymptote is approached very quickly in the case of binomial and multinomial distributions. Following the above functions, we can compute the likelihood ratio for each label, which determine the rank in a cluster.
