# Supplementary material for: Characteristics and Emerging Trends in Research on Rehabilitation Robots from 2001 to 2020: Bibliometric Study
Source: J Med Internet Res. 2023 May 31;25:e42901. doi: 10.2196/42901 (PMC10267796; doi:10.2196/42901)
Supplement: Multimedia Appendix 2 [file jmir_v25i1e42901_app2.docx]

**The burst detection algorithm and its interpretation**

The burst detection is an important analysis in the bibliometric study, which has been used to explore the development of a research field over time. In this study, the burst detection was determined using an algorithm reported by Kleinberg et al [44]. Originally developed to detect topics in email chains, Kleinberg’s algorithm defines the burst with an infinite state automaton in which each state represents a message arrival rate. The higher the state, the smaller the expected time gap between messages [45]. The burst then can then be defined as having arrival rates defined by the number of messages containing a particular word. Additionally, jumping from a lower state to a higher state has an associated cost, while the cost to drop down from a higher state to a lower state is 0. Formally, these states are determined by optimization:

for time series

$z=\left\{ z_{t} | t=0,1,\ldots,n \right\}$

find a state sequence

$$q=\left\{ q_{i_{t}} | t=0,1,\ldots,n \right\}$$

to minimize cost function

$$c\left( q | z \right)=b\left( q \right)\ln((1-p)/p)+\left( \sum_{t=0}^{n} -\ln f_{i_{t}}(z_{t}) \right)$$

where *p* is the probability of a state change, *b*(*q*) is the number of state transitions (changes in successive states) in *q*, and $f_{i}\left( z \right)=\alpha_{i}e^{-\alpha_{i}z}$ is the exponential density function for gap values *z* with arrival rate $\alpha_{i}$.

By finding the optimal sequence of states minimizing the cost of transitions and the cost of differences between real arrival rate and the predicted emission rate, a time series of burst strengths is obtained. Therefore, the value of burst strength is the relative value which is greater than zero. Greater values of the burst strength, in our study, indicate a sharper increase of specific keywords in citations of “rehabilitation robot” literatures during a period.
